# Associations between anthropometric characteristics and physical performance in male law enforcement officers: a retrospective cohort study

**DOI:** 10.1186/s40557-016-0112-5

**Published:** 2016-06-10

**Authors:** James Jay Dawes, Robin Marc Orr, Claire Louise Siekaniec, Andrea Annie Vanderwoude, Rodney Pope

**Affiliations:** Department of Health Sciences, University of Colorado-Colorado Springs, Colorado Springs, CO USA; Tactical Research Unit, Bond University, Robina, Queensland Australia; Gatorade sports nutrition intern, University of Southern California, Los Angeles, CA USA

**Keywords:** Police, Body fat, Fitness, Strength, Tactical

## Abstract

**Background:**

Police officers are often required to undertake physically demanding tasks, like lifting, dragging and pursuing a suspect. Therefore, physical performance is a key requirement.

**Methods:**

Retrospective data for 76 male police officers (mean age = 39.42 ± 8.41 years; mean weight = 84.21 ± 12.91 kg) was obtained. Data included anthropometric (skinfolds, estimated percentage body fat, lean body mass and fat mass) and physical performance (1 Repetition Maximum Bench Press, 1–min sit-ups, 1-min push-ups, vertical jump, 300 m run, 1.5 mile run) measures and correlations between anthropometric measurement and fitness score were obtained.

**Results:**

Estimated percentage body fat was significantly (*p* ≤ .001) and negatively correlated with all performance measures, except sit-ups and 300 m and 1.5 mile run performance. Estimated lean body mass was significantly and positively (*p* ≤ .001) correlated with push-ups, bench press and vertical jump measures, while increasing estimated fat mass was significantly (*p* ≤ .001) associated with reduced performance on sit-up, vertical jump, 1.5 mile run and estimated maximal voluntary oxygen uptake.

**Conclusions:**

A targeted approach, going beyond just decreasing percentage body fat to also selectively increasing lean mass, should be applied for optimal improvement in physical fitness performance.

## Background

At any point in time, on-duty law enforcement officers may be required to push, pull, lift, carry, drag, jump, vault, crawl, sprint, use force, and sustain pursuit of a suspect [1–3]. Therefore, physical fitness is a key requirement for this population. Although there are no national standards of physical fitness for law enforcement officers, The Cooper Institute of Aerobic Fitness [4] recommends a battery of tests for this population that includes 1-min sit-up and 1-min push-up tests to assess muscular endurance, a 1 repetition max (RM) bench press (BP) to assess muscular strength, a 300 m run and vertical jump test to assess anaerobic power, and a 1.5 mile run to assess aerobic fitness. Although these fitness tests cannot serve as a complete predictor of overall fitness and job performance, empirical research has shown a link between levels of fitness and risk of injury [5, 6]. Furthermore, levels of fitness, and in turn cardiovascular health, are known to be related to the risks of cardiovascular disease; a recognized cause of mortality in police officers, with police officers twice as likely as the general population to suffer this disease [7]. On this basis, means of improving, or at least maintaining, levels of fitness are important.

Anthropometric measurements, like skinfold measurements, are often used as a predictor of performance and health status in the general population [8]. While anthropometric measurements may not be suitable as a determinant of law enforcement selection they may be useful in predicting physical capability, job performance and, as such, identifying areas for improvement, particularly at a cohort level. Considering this, limited research has been done in tactical populations (like firefighter/first responder, military and law enforcement) on the use of skin fold anthropometric measurements, with the results suggesting a relationship between skinfold thickness and physical performance.

Williford and Scharf-Olson [9] demonstrated a relationship between sum of skinfolds and job performance in firefighters. Overall, the researchers found that a higher percentage of body fat was linked with poorer performance in simulated job tasks and could be used as a predictor of job performance. Similarly, Ricciardi, et al. [10] observed a reduced aerobic capacity and load carriage task performance ability in military participants with increased levels of body fat. Even when participants were wearing relatively light loads (10 kg body armour), the amount of body fat of males (17 %) and females (26 %) was found to negatively correlate (*r* = −0.88; *p* < .001) with physical task performance [10]. In a recent study of a law enforcement population, Dawes et al. [11] found that the sum of skinfolds, as recommended by Jackson and Pollock, was negatively correlated with muscular endurance assessments (push-ups, sit-ups, pull-ups) as well as obstacle course performance in Special Weapons and Tactics (SWAT) officers. The strongest significant relationship occurred between sum of skinfolds and pull-up assessment. While these reported relationships between fat mass and physical fitness performance do not provide conclusive evidence of cause-and-effect relationships, they do suggest that physical training to decrease overall fat mass may, either directly (reducing the non-functional mass to be lifted) or indirectly (muscular training effect) have a positive impact on muscular endurance performance. These reported relationships therefore warrant further scrutiny.

Not all studies support the findings of poorer physical performance with increased levels of body fat. Both Frykman, et al. [12] and Pandorf, et al. [13] found that body fat (21-32 %) did not affect assessed performance of load carriage tasks like obstacle courses and a 3.2 km loaded run. However, whether this finding reflected body fat alone, or the influence of overall body weight and stature on absolute VO2max, was not apparent. Given the contrasting findings, and the reliance that society places upon these tactical personnel having the physical capability to perform their jobs efficiently and effectively, further research, specifically investigating associations between body composition and physical performance in a law enforcement population may be of benefit. Therefore, the purpose of this study was to investigate and report on the relationship between body composition (as determined through skinfold measurements) and physical fitness performance in male law enforcement officers.

## Methods

### Participants

Retrospective data for seventy-six (*n* = 76) male law enforcement officers (mean age = 39.42 ± 8.41 year; mean weight = 84.21 ± 12.91 kg; mean est. VO2 max = 41.31 ± 65 ml/kg/min) belonging to a volunteer fitness program were analysed in this investigation. Approval was obtained from the University of Colorado, Colorado Springs Institutional Review Board for human subjects (IRB 15-074) and the Bond University Human Research Ethics Committee (RO1927), prior to the analysis of this retrospective data.

### Measures

All anthropometric measures (weight and 3-site skinfold measurements) and muscular strength, power and endurance assessments (1 RM Bench press, 1 –min sit-ups, 1-min push-ups, vertical jump) had been conducted indoors at the law enforcement agency’s training facility. Measures of anaerobic (300 m) and aerobic (1.5 mile run) fitness had been performed with participants running around a predetermined course spanning local city blocks as fast as possible. Assessment protocols and order used when the retrospective data were collected are listed below.

#### Weight

Weight (lbs) measurements for the officers were collected using standard procedures on a doctors beam scale (Cardinal; Detecto Scale Co, Webb City, MO), and then entered into a spreadsheet and converted to kgs.

#### Skinfold measurements

Employing the skinfold assessment protocol as recommended by Jackson and Pollock [14] and previously employed in a law enforcement population [11], sum of skinfolds (SS) were collected from three sites. Duplicate measures of the chest, abdomen, and thigh were taken on the right side of the body using Lange Skinfold Calipers (Lange, Beta Technology Inc, Cambridge, MD), rotating among sites to allow skin to regain normal texture and thickness, and recorded to the nearest centimeter. Percentage body fat (%BF), lean body mass (LBM [kg]) and fat mass (FM [kg]) were calculated using scale weight and the prediction formula provided by Jackson and Pollock [15].

#### 1-min push-ups

Upper-body muscular endurance was measured using a protocol previously employed in a law enforcement population [11] with the duration modified to 1-min. All officers were required to begin the test with the body rigid and straight, the elbows fully extended, the hands positioned slightly wider than shoulder-width apart and the fingers pointed forward. This position constituted the ‘up’ position. To facilitate and control push-up depth, a partner placed a closed fist on the floor directly under the officer’s chest. On the verbal command ‘go’ the participant proceeded to bend their elbows, lowering themselves until their chest was in contact with their partner’s fist and then extending their elbows until back in the ‘up’ position. The officer continued in this fashion repeating as many repetitions as possible within the 1-min period recorded by the tester on a handheld stopwatch. Officers were allowed to rest in the straight-arm position, as long a neutral trunk position was maintained and the time had not elapsed. The test was terminated when an officer was unable to perform this movement with proper technique, or when the 1-min time limit expired.

#### 1-min sit-ups

The technique used for this test is detailed by Hoffman and Collingwood [16]. All officers were required to begin the assessment lying in a supine position with the knees bent to around 90° and the feet flat on the ground. Hands were placed behind the neck with fingers interlaced. Once in position the participant flexed their trunk, elevating their shoulders off the floor until their elbows touched their knees. During this assessment each officer had a partner anchor their feet in place to assist in keeping the feet flat on the floor throughout the exercise movement. On the verbal command ‘go’ the tester began the stopwatch and the participant began the assessment. The officers then proceeded to perform as many sit-ups as possible in 1-min using this technique.

#### Vertical jump

Vertical jump height was collected using a Vertec™ apparatus (Vertec Scientific Ltd., Aldermaston, UK). After determining the standing upward reach height for each officer they were instructed to perform a rapid countermovement jump with an arm swing, jump as high as possible, and attempt to displace the horizontal plastic fins on the device. The best of three attempts was taken and maximal jump height was recorded to the nearest 1.2 cm (0.5 in.). Peak power output was then calculated using the Sayers power equation (See Equation 1) [17]. This equation is considered to be more valid than that of Harman et al [18] in estimating peak power from vertical jump [17].

Equation 1: Sayers [17] Peak Power equation

$$ Peak\  power\ (Watts)=60.7\times height(cm)+45.3\times body\  mass(kg)-2055 $$

#### 1 RM bench press

Officers were instructed to begin by lying down in a standard bench press rack and positioning themselves on the flat bench. Officers were then instructed to maintain a 5-point contact (head, shoulders and glutes in contact with the bench and both feet on the floor). Eyes were lined up directly below the barbell on the rack. The barbell was then lifted off the rack until it was positioned directly over the officer’s chest. In a controlled manner, the officer lowered the bar to the chest, lightly touched the bar against the chest, then extended the arms to return the bar to the starting position. This 1RM was then converted to a 1RM Bench press ratio (BPR) score (weight lifted/body weight) in order to measure relative upper-body strength.

#### 300 meter run

A 300 m course was measured around a city block. Officers were instructed to run this course, as fast as possible. Upon completion each participant’s time was recorded to the nearest 0.10 s on a hand held stopwatch.

#### 1.5 mile run

A ¾ mile course was measured around local city blocks. Following a 2 h rest, officers were instructed to run this course twice, as fast as possible. Upon completion each participant’s time was recorded to the nearest 0.10 s on a hand held stopwatch. Estimated VO2 max was then calculated using charts provided by the Cooper Institute for Aerobics Research [19].

### Statistical analysis

The data was entered into a computer file suitable for statistical analysis using the SPSS 22.0 software package [20]. A descriptive statistical analysis was conducted to determine mean scores and standard deviations for each skinfold site and each measure of performance. Participant results were summarized for two participant groupings, based on anthropometric results. These groups were: (i) those participants found to have an ‘above average’ percentage body fat for their age group; being above 15.0 % for males up to the age of 30 years of age, and above 17.0 % for males up to 50 years of age; and (ii) ‘average’ percentage body fat and below, being 15.0 % and below for males up to the age of 30 years of age, and 17.0 % and below for males up to 50 years of age [8]. These groups were devised from normative data in order to allow for future simplified categorizations through which to implement potential recommendations. Differences between these groups in physical performance on the measured fitness characteristics were first determined using an independent samples *t*-test, and subsequently using an ANCOVA with age included as a covariate. Pearson’s correlation analyses were performed to investigate relationships between the anthropometric and performance measures. The level of statistical significance was set *a priori* at 0.001. This stringent level of significance was chosen in order to control the family-wise error rate (or probability of making spurious significant findings, or Type I errors, when conducting multiple statistical tests of hypotheses), that would otherwise accompany the large number of statistical tests performed [21].

## Results

Descriptive results for actual and estimated anthropometric measurements and actual and estimated fitness performance results for the cohort as a whole and for each of the two body fat groups are presented in Table 1. Significant differences between the two groups are also indicated in Table 1, based on the results of independent samples *t*-tests.

**Table 1:** Descriptive information for full-time officers as a cohort and by %BF groupings

Measure	Cohort Mean ± SD	‘Average’ and below group^b^ Mean ± SD	‘Above average’ group^b^ Mean ± SD
	*n* = 76	*n* = 31	*n* = 45
Weight (kg)	84.45 ± 12.80	82.82 ± 13.40	85.58 ± 12.40
Chest skinfold (mm)	13.74 ± 5.52	8.77 ± 3.54	17.16 ± 3.77^a^
Abdominal skinfold (mm)	24.57 ± 8.85	17.74 ± 6.52	29.27 ± 7.00^a^
Thigh skinfold (mm)	12.72 ± 4.99	10.19 ± 3.26	14.47 ± 5.25^a^
Sum of skinfolds (mm)	51.01 ± 14.56	36.71 ± 9.03	60.87 ± 7.88^a^
Estimated body fat (%)	16.89 ± 4.60	12.40 ± 3.21	19.98 ± 2.25^a^
Estimated lean mass (kg)	70.21 ± 11.45	72.71 ± 12.82	68.48 ± 10.20
Estimated fat mass (kg)	14.24 ± 4.50	10.11 ± 2.66	17.09 ± 3.06^a^
Push-ups (reps)	55.58 ± 17.35	64.39 ± 16.39	49.51 ± 15.43^a^
Sit-ups (reps)	41.05 ± 6.96	43.51 ± 6.34	39.32 ± 6.92
Vertical jump height (cm)	61.26 ± 7.96	65.75 ± 7.55	58.17 ± 6.71^a^
Estimated peak power (w)	5478.38 ± 829.96	5661.33 ± 828.93	5352.34 ± 815.99
Bench press (kg)	93.79 ± 25.91	102.21 ± 27.16	88.00 ± 23.60
Bench press ratio (BPR)	1.10 ± 0.23	1.22 ± 0.23	1.02 ± 0.18^a^
300 m (secs)	56.03 ± 10.67	52.96 ± 6.26	58.15 ± 12.49
1.5 mile run (min:secs)	12.75 ± 2.30	11.86 ± 1.47	13.37 ± 2.57
Estimated VO2 max (ml.kg.min.-1)	41.31 ± 6.50	43.96 ± 4.36	39.49 ± 7.12

^a^Significant difference between groups, with *p* < .001

^b^Groups: i) ‘Average and below’ included participants with a %BF of 15.0 % and below for males up to the age of 30 years of age, and 17.0 % and below for males up to 50 years of age, and ii) ‘Above average’ included participants with a %BF above 15.0 % for males up to the age of 30 years of age, and above 17.0 % for males up to 50 years of age [8]

The ANCOVA (Table 2) indicated that age was not a significant predictor of any of the performance variables, when the level of significance was set at 0.001 as planned. While participants with an above average %BF were observed to consistently perform at a lower level on physical fitness tests than those with average or below average %BF (Table 1), this difference only reached statistical significance (*p* ≤ .001; Tables 1 and 2) in push-ups, vertical jump height and bench press ratio.

**Table 2:** ANCOVA results between groups with age as the covariate

		Age	%BF groups
*n*	Measure	*F* value and Sig	*F* value and Sig
76	Push-ups (reps)	*F*_(1,73)_ = 0.282, *p* = .597	*F*_(1,73)_ = 15.113, *p* < .001^a^
76	Sit-ups (reps)	*F*_(1,73)_ = 3.100, *p* = .083	*F*_(1,73)_ = 8.674, *p* = .004
76	Vertical jump height (cm)	*F*_(1,73)_ = 10.299, *p* = .002	*F*_(1,73)_ = 19.018, *p* < .001^a^
76	Estimated peak power (w)	*F*_(1,73)_ = 1.536, *p* = .219	*F*_(1,73)_ = 2.013, *p* = .160
76	Bench press (kg)	*F*_(1,73)_ = 0.243, *p* = 0.623	*F*_(1,73)_ = 5.354, *p* = .023
76	Bench press ratio (BPR)	*F*_(1,73)_ = 1.339, *p* = 0.251	*F*_(1,73)_ = 17.119, *p* < .001^a^
75	300 m (secs)	*F*_(1,73)_ = 2.067, *p* = .155	*F*_(1,73)_ = 3.661, *p* = .060
76	1.5 mile run (min:secs)	*F*_(1,73)_ = 3.121, *p* = .081	*F*_(1,73)_ = 7.270, *p* = .009
76	Estimated VO2max (ml.kg.min.-1)	*F*_(1,73)_ = 1.755, *p* = .189	*F*_(1,73)_ = 8.388, *p* = .005

*%BF* Percentage Body Fat

^a^significant difference at *p* < .001

Table 3 demonstrates the correlations between specific anthropometric measurements and specific physical fitness test results. Consistent with the findings reported from the independent samples *t*-tests (Table 1) and ANCOVA (Table 2), comparing groups based on %BF, the Pearson’s correlation analyses indicated that body composition, assessed in terms of %BF, was significantly correlated with push-ups, vertical jump height, bench press ratio and estimated VO2max (Table 3). However, there were differences noted in the associations between estimated lean mass (LM; kg) and estimated fat mass (FM; kg) and physical fitness performance measures. LM was significantly and positively correlated with push-ups, vertical jump height, estimated peak power, bench press and bench press ratio, while increasing FM was significantly associated with reductions in performance on vertical jump, 1.5 mi run and estimated VO2max (Table 3).

**Table 3:** Correlations between anthropometric measures and fitness scores

Fitness and anthropometric information	%BF	LM (kg)	FM (kg)
Push-ups (reps)	−.413^a^	.444^a^	−.210
Sit-ups (reps)	−.198	−.177	−.308
Vertical jump height (cm)	−.566^a^	.391^a^	−.369^a^
Estimated peak power (w)	−.343	.879^a^	.107
Bench press (kg)	−.327	.781^a^	.073
Bench press ratio (BPR)	−.448^a^	.392^a^	−.241
300 m (secs)	.244	.049	.290
1.5 mile run (min:secs)	.285	.181	.399^a^
Estimated VO2 Max.(ml.kg.min.-1)	−.287	−.214	−.419^a^

*%BF* Percentage Body Fat, *LM* Lean Mass, *FM* Fat Mass

^a^Correlation is significant with *p* ≤ .001

## Discussion

The purpose of this study was to investigate the relationship between body composition (as determined through skinfold measurements) and physical fitness performance in male law enforcement officers. In general, the results suggest that body composition, when assessed in terms of %BF or FM (measured in kg) or LM (also measured in kg), is associated with physical fitness performance. Apart from the intuitive association between higher %BF and its associated poorer performance, the results go further in suggesting that FM may not be strongly associated with all forms of physical performance and rather decreased LM may be more strongly associated with, and possibly responsible for decreases in some physical fitness performance measures. In essence, the study found that for measures of aerobic fitness, FM may be of greater predictive importance than LM and, conversely, for measures of strength and muscular endurance (like bench press, peak power and push-ups), LM may be more important than FM.

There were two exceptions—the sit-up and the vertical jump. Unlike the other measures of muscular endurance, sit-up performance was more strongly correlated with FM than LM, though it should be noted that the negative relationship between sit-up performance and FM did not quite reach the stringent level of statistical significance adopted in this study (Tables 2 and 3). A potential reason for this difference is the distribution of fat mass, with previous research [11] suggesting that an increased sum of skinfolds around the abdomen impacted on sit-up performance but not push-up performance.

The other exception was that of vertical jump height which was significantly correlated with both FM (negative correlation) and LM (positive correlation). A potential reason for this finding is that the nature of the vertical jump requires an element of strength but also the ability to lift the entire body mass off the ground (like running). As such, although the expectation that increased LM would increase performance on this power task held true (ie the increased LM appeared to generate a sufficient increase in leg power to overcome and exceed the additional LM to be lifted), it was also notable, though not unexpected, that increased FM had the opposite effect.

This information is particularly useful in suggesting ways in which performance improvement might be achieved in the law enforcement population. The correlations between lean mass and performance measures were stronger in strength, muscular endurance and power orientated aspects of performance than correlations of those performance measures with fat mass. It may therefore be more effective to intervene with training designed to increase lean muscle mass and muscle strength, endurance and power rather than work primarily to decrease fat mass in order to improve strength, endurance and power related physical fitness performance. Conversely, focusing on decreasing fat mass and increasing metabolic fitness and muscular endurance, rather than increasing lean body mass, may be the most appropriate approach when attempting to increase run times, be they anaerobic or aerobic in requirement. It is important, that other factors that influence performance, like current sport and exercise regime, smoking and diet are likewise considered.

The limited available studies profiling and investigating the anthropometric and fitness characteristics of police officers restricts the ability to compare the findings of this study to results of other studies. However, from the limited studies available, the participants in this study, while of similar ages, were found to be generally lighter (84.45 ± 12.88 kg) than those observed in two separate studies by Boyce et al. (93.2 ± 16.2 kg [22]: 94.6 ± 15.9 kg [23]) and have a lower %BF (16.9 ± 4.6 compared to 19.1 ± 5.9 [22] and 18.5 ± 6.2 % [23] respectively). One of the studies by Boyce et al, [23] reported measures of FM and LM and while the participants in this study had less FM (14.25 ± 7.50 kg versus 18.7 ± 8.5 kg) than participants in the study reported by Boyce et al. [22], they also had less LM (70.21 ± 11.45 kg versus 75.9 ± 9.6 kg). The other study by Boyce et al. [22] included one measure of strength, the bench press, in which their participants recorded slightly higher scores (96.3 ± 20.9 kg) than those observed in this study (93.79 ± 25.91 kg). When considering the %BF and weight of the participants in this latter study by Boyce et al., [22], the higher absolute bench press results align with the findings of this study which indicated a significant and strong correlation between LM and bench press (kg) results.

### Limitations

The data analyzed for this study was retrospective and therefore some data of value was missing. For example, it would have been beneficial to include participant height data in the analyses, but this was unavailable for this cohort. In addition, only three skinfold sites were used, and future research may benefit from the use of 7 skinfold sites [24] to increase skinfold sensitivity and as such the influence of skinfold site measures at specific sites and performance. Finally, the participants in this study were volunteers undertaking a fitness program as such there may be limitations in generalization to the broader population. Considering this, while there is a potential for the data to have excluded the very unfit who may not have wanted to participate in this program, the opposite may be true whereby the very fit would follow their own current fitness regime.

## Conclusion

In conclusion, physical performance in law enforcement is critical and anthropometric measurements can be used to guide conditioning interventions to improve performance. This study supports evidence that increasing %BF is associated with decreasing performance. More importantly, this study suggests a targeted approach, going beyond just decreasing %BF to also selectively increasing lean mass, should be applied in efforts to achieve optimal improvement in physical fitness performance. For example, rather than reducing body fat, increasing lean body mass should be the conditioning goal to increase performance in upper-body muscular endurance measures (like push-ups and bench press) and peak power generation. Conversely, decreasing body fat and at the same time improving metabolic fitness and muscular endurance should be the conditioning goal to improve sit-up performance and run times, be they short or longer distance. Increasing lean body mass and decreasing body fat can both positively influence vertical jump performance.
